# Urinary diseases and ethnobotany among pastoral nomads in the Middle East

**DOI:** 10.1186/1746-4269-1-4

**Published:** 2005-08-02

**Authors:** Aref Abu-Rabia

**Affiliations:** 1 Department of Middle East Studies, Ben-Gurion University, Beer-Sheva, 84105, Israel

## Abstract

This article is derived from a broad, twenty-year study of ethnobotany and folk medicine among pastoral nomads in the Middle East which took place from 1984 to 2004. The article presents examples of different treatments of diseases and disorders of the urinary tract carried out by healer herbalists. The preparation of remedies includes boiling infusions, extraction of dry or fresh leaves, flowers, seeds or whole plants. Some of these plants were used both as food and as medicine, by ingesting different parts of the plants, such as leaves, flowers, fruits, and so on, either while soft, cooked or dried. Data were collected by using unstructured interviews and by observation. These plants were identified by healers, patients, and university botanists. This paper identified eighty-five plant species, which belong to thirty-six families. The most representative families are: Asteraceae (8), Brassicaceae (6), Poaceae (6), Umbelliferae (6).

## Introduction

People have been using traditional medicine including ethno-botany for several thousand years. Ancient Arabic medicine was influenced by the ancient medicinal practices of Mesopotamia, Greece, Rome, Persia and India. The Greco-Roman system of medicine was developed based primarily on the writings of Hippocrates (460-360 B.C.), Dioscorides (circa 54 to 68 AD) and Galen (130–201 AD). A combination of political and religious factors caused many Greek and Syriac-speaking scholars to move eastward to Persia and to establish centers of learning there. The city of Gundishapur in southwest Iran also became a center of learning, with a well-known medical school, in the sixth century AD [1,2]. One of the Arab physicians during the time of the Prophet Muhammad (571–632 AD) was al-Harith ibn Kalada (d. 634), one of the most prominent physicians of his time, who traveled to Gundishapur in Persia and studied medicine prior to the establishment of Islam. Another renowned Arab physician was Ibn Abi Rimtha. The sayings (*Hadith*) of the Prophet Muhammad on health and illness were systemized and became known as *The Medicine of the Prophet *(*al-Tibb al-Nabawi*) [3,2]. During the Umayyad rule (from 661–750 in the East, based in Damascus), many ancient medical works began to be translated. For five centuries (750–1258) the Abbasids, based in Baghdad, dominated the socio-political life of the greater part of the Muslim world. Countless manuscripts, particularly those written in Greek, were collected and stored in *Bayt al-hikmah *(The House of Wisdom, established in 830, by the Caliph al-Ma'mun), where scholars worked to translate them into Arabic [4,5].

Within a century, Muslim physicians and scientists were writing original contributions to medical and botanical knowledge. One of the greatest and most famous Islamic doctors was Ibn Sina (Avicenna 980–1037), author of *The Canon of Medicine *(*Kitab al-Qanun fi al-Tibb*), the epitome of Islamic medicine. This work is the culmination and masterpiece of the Arab systematization of medical science, and includes many descriptions of the uses of medicinal plants [6]. Other Arabic philosopher-physicians were al-Razi (Rhazes 865–923) who wrote *The Comprehensive Book on Medicine *(*Kitab al-Hawi fi al-Tibb*). The material written by al-Hawi is arranged under headings of different diseases, with separate sections on pharmacological topics. Ibn Sina's and al-Razi's works were later translated into Latin, and continued to influence medical science well into the nineteenth century [7-9].

In the western part of the Islamic empire, the Umayyads of Andalus (Islamic Spain) made their capital at Cordoba. Areas of Cordoba and Granada became centers of learning. The richness and diversity of the flora of Spain were major contributing factors to the development of medical botany. The majority of physicians were herbalists and vice versa. The physician Ibn al-Baytar (1197–1248), authored *The Compendium of Simple Drugs and Food *(*al-jami' li-mufradat al-adwiya wa'l-aghdhiya*), in which he described more than 1400 medicinal drugs, 300 of which had not previously been described, recording them alphabetically and discussing them with great clarity and detail. The work specified the names of herbs and remedies in various languages, thus providing a first class tool for the comparative research of medicinal plants. Other well-known physicians who wrote on plant uses were: Ibn Juljul, al-Ghafiqi, Ibn Bajjah, Ibn Samajun, and Abu'l-Hassan al-Andalusi [10,7]. Traditional medical information grounded in the Arab medicine of the Middle Ages was gradually transferred to traditional healers and to the general public [11]. The use of herbal medicine is still widespread throughout the populations of the Middle East, including the pastoral nomadic tribes [12-19].

Among the pastoral Bedouin, hundreds of species of trees and shrubs are employed as analgesics, astringent, diuretics, emetics, purgatives, poultices, salves, and tonics. Some of these herbs are aimed at cleansing the pastoralist's body of polluting influences, bad spirits, *jinns*, and the negative effects of sorcery and/or witchcraft. The pastoral nomadic tribes depend on their local healers and traditional medicine as recorded in Table 1 (see Additional file 1).

## Methodology

The data for this paper are derived from a broad twenty-year study of ethnobotany and folk medicine among the pastoral nomadic Bedouin tribes in the Negev, Jordan and Sinai deserts, carried out from (1984–2004). The paper is based on interviews with healers and patients. All the material was recorded in field logs, and some was tape-recorded. Unstructured interviews and the observation of participants were carried out in the informants' homes (120 men and 120 women), as well as in the homes of traditional healers (15 men and 10 women). Most of the healers were in the age range of forty to eighty. All the informants were married and over thirty. There were five males from each desert, and four female healers from the Negev, three from Sinai and three from Jordan. The informants were divided into two groups of forty men and forty women from each desert. The collected information was used to construct a list of the indigenous ethnobotanic medicine. Samples from all the plants were collected and identified by healers, patients and university botanists.

## Results and Discussion

This paper describes the treatment of diseases and disorders of the urinary tract by traditional herbalists among the pastoral nomadic Bedouin tribes in the Middle East. In this study, we identified eighty-five plant species, which belong to thirty-six families.

The use of traditional medicine by the pastoral nomads, and the appeal to traditional healers over the course of many centuries established a psychological-therapeutic dependence of the pastoral nomadic tribes upon these healers. The rich variety of approaches employed by pastoral nomadic healers to treat disorders and diseases of the urinary tract is indicative of the depth and breadth of indigenous medicine practiced among the pastoral nomads in the twentieth century. The analysis of my collected data, together with the information extracted from the literature on herbal and ethnobotanic medicine of countries in the Middle East [14,20,15,21,18], yielded Table 1 (see Additional file 1). This table includes eighty-five plants with medicinal potential which have been used among the pastoral nomadic Bedouin tribes in the Middle East from generation to generation as reported by my informants.

Table 1 presents information on which parts of the plants are used and in what manner. It should be noted that for some plants, the uses in different countries of the Middle East are similar [22,16]. However, dissimilar uses were also observed for certain plants in different countries/tribes in the Middle East [23,15,19,24]. The important information gathered in this study will help to preserve the heritage and knowledge of ethnobotanic and folk medicine of the indigenous pastoral tribes of the Middle East. This study will generate awareness in the region concerning the potential for conserving plant resources in medicine, food, nutrition and folk heritage. It is of the utmost importance to preserve this heritage, which relates to the traditional, economic and medicinal uses of available plant resources in the countries of the Middle East.

The many medicinal substances which we were able to identify as used in traditional medicine included various plants species. The analysis of the findings shows that the three deserts where I conducted my research served as the geographic origin of the medicinal substances. These plants were available because they grew as wild and cultivated plants and were part of the natural flora of these deserts. The pastoral nomads used these plants as food and as medicine, by eating different parts of the plants including the following: leaves, flowers, barks, stems, stalks, roots, rhizomes, bulbs, pith, fruit, corms, inflorescenes, shells, berries, seeds, stones/pits (in fruit), soft seed pods, buds, and shoots.

It should be noted that wild desert plants also contain a host of other biologically active compounds besides nutrients. The physiological effects of these other compounds in relation to plant nutrients are not well known, but could affect nutrient and medical utilization or other functions. These topics are of relevance for future research in terms of improving our understanding of human nutritional and medical requirements of the pastoral nomads in the Middle East.

## Supplementary Material

Additional file 1 Table 1: Urinary Diseases and Ethnobotany among Pastoral Nomads in the middle East Click here for file
