# Supplementary material for: Urinary diseases and ethnobotany among pastoral nomads in the Middle East
Source: J Ethnobiol Ethnomed. 2005 Aug 2;1:4. doi: 10.1186/1746-4269-1-4 (PMC1277084; doi:10.1186/1746-4269-1-4)
Supplement: Additional file 1 — Table 1: Urinary Diseases and Ethnobotany among Pastoral Nomads in the middle East [file 1746-4269-1-4-S1.doc]

# Table 1: URINARY DISEASES AND ETHNOBOTANY AMONG PASTORAL NOMADS IN THE MIDDLE EAST

| Scientific family | Scientific name | Local name | Plant parts | Preparation | Ethno-botanical uses |
| --- | --- | --- | --- | --- | --- |
| **Adiantaceae** | Adiantum capillus-veneris L | *Kuzbarat al-Beir, Sha'arat al-Ghulih* | leaves and roots | boil in water and drink | Colds, diuretic, urinary tract infections |
| **Anacardiaceae** | Rhus coriaria L | *Summaq* | fruit | drink extracted liquid from the fruit | urinary tract infections and urinary retention |
| **Boraginaceae** | Anchusa strigosa Banks et Sol. | *Ihmim, lisan al-thur* | leaves and roots | crush, soak in water, and drink | urinary tract infections |
| **Caesalpiniaceae** | Cassia italica (Mill.) Lam. Ex F.W. Andrews | *Kasiah, Sinamakky* | leaves | boil in water and drink | urinary retention |
|  | Cercis siliquastrum L. | *‘Arus al-Ghaba* | leaves and flowers | boil in water and drink | urinary retention and infections |
| **Capparaceae** | Capparis spinosa L. | *Qubbar, Lasaf* | bulbs | soak in water and drink | diuretic, urinary tract infections and retention |
| **Caryophyllaceae** | Paronychia argentea Lam. | *Rijil al-Hamamih, Rijil al-'Asfur* | leaves and flowers | boil in water and drink | diuretic, urinary retention and urinary tract infections |
| **Chenopodiaceae** | Beta vulgaris L. | *Shamandar, salq, banjar* | leaves and roots | raw | urinary tract infections |
|  | Chenopodium album L. | *Fisa al-Kalb* | the whole herb | soak in water and drink | urinary tract infections and retention |
| **Asteraceae** | Cichorium pumilum Jacq. | *'Iltt, 'Ilk*, *hindabah* | green leaves | eat, or soak in water and drink | diuretic, urinary tract infections and retentions |
|  | Cnicus benedictus L. | *Kassuba* | leaves, stalks and roots | boil in water and drink | urinary retention and urinary tract infections, prostate illness |
|  | Inula viscose (L.) Ait. | *‘Irq al-Tayun, Tayun* | leaves | boil in water and drink | urinary retention and urinary tract infections |
|  | Matricaria aurea (Loefl.) Sch. Bip. | *Babunaj*, *Rabil* | leaves and flowers | boil in water and drink | urinary retention, urinary tract infections, kidney pains, testicle pain |
|  | Phagnalon rupestre (L.) DC. | *Qadih,Sufan Qray’i* | leaves and stalks | boil in water and drink | urinary retention and urinary tract infections |
|  | Scolymus maculatus L. | *Sinariya Hawliya*), | stems, stalks | boil in water | urinary tract infections |
|  | Taraxacum megalorrhizon (Forssk.) Hand.-Mazz. | *Salatat al-Ruhban* | Flowers and leaves | eat as raw salad | urinary retention and urinary tract infections |
|  | Varthemia iphionoides Boiss. et Bl. | *Slimaniya, Ktilih* | Leaves and stalks | boil in water and drink | urinary retention and urinary tract infections |
| **Brassicaceae** | Anastatica hierochuntica L. | *Kaf al-Rahaman, Kaff Maryam* | dried fruit | crush the fruit, soak in water and drink | urinary retention and urinary tract infections, female sterility, male impotency |
|  | Brassica nigra (L.) Koch | *Left* | stems, seeds | eat or soak in water and drink | urinary retention and urinary tract infections, stomach ache |
|  | Capsella bursa-pastoris L. Medik. | *Kis al-Ra’y* | dried leaves | boil in water and drink | urinary retention and urinary tract infections |
|  | Lepidium sativum L | *Habb al-Rashad, Hurf* | seeds | boil in water and drink | urinary retention and urinary tract infection, prostate illnesses |
|  | Raphanus raphanistrum L. | *Fijil* *barri*, *Fijjilih* | leaves | boil the leaves in water and drink | urinary tract infections and urinary retention |
|  | Sisymbrium irio L. | *Kibs* | leaves and flowers | eat as raw salad | urinary retention and urinary tract infections |
| **Cucurbitaceae** | Citrulus vulgaris Schirad | *Battykh* | fruit | eat | urinary tract infections |
|  | Cucumis melo L. | *Shimmam* | fruit | eat | urinary retention and urinary tract infections, kidney pains, constipation |
|  | Cucumis sativa L. | *Khiar, Khyar* | fruit | eat | urinary retention and urinary tract infections |
|  | Ecbalium elaterium (L.) A. Rich. | *Qitha al-Hameir, fagus al-Hameir* | fruit, seeds | squeeze or soak in water and drink | urinary retention and urinary tract infections, yellow fever, liver infections |
| **Cupressaceae** | Juniperus communis,  Juniperus oxycedrus L. Juniperus phoenicea L. | *'Ar'ar, ‘Itran, Qitran* | berries | soak in water and drink | diuretic, urinary retention and urinary tract infections, kidney pains, prostate, impotency, female sterility, vaginal infection. |
| **Globulariaceae** | Globularia arabica Jaub. et Spach | *'Aynun* | leaves, flowers | soak in water and drink | urinary incontinence and urinary tract infections, kidney pains |
| **Poaceae** | Elymus repens (L.) Gould | *Nejil, Njil* | leaves and roots | boil in water and drink | urinary tract infections, and cystitis |
| **Poaceae** | Hordeum sativum Hordeum spontaneum C. Koch.  Avena sterilis L. | *Sha’ir*  *khafour, shufan* | seeds, bran  Leaves, seeds | eat as pita, boil crushed seeds in water and drink  eat bread or prepare porridge | urinary tract infections and retention  urinary retention and infections, diabetes, increasing breast milk and strengthening mother body after childbirth |
|  | Triticum aestivum L. | *Qamh*, hintta | Leaves, seeds, bran | eat as pita, soak seeds or bran in water and drink | urinary retention and prostate illnesses, impotency, diabetes, high blood pressure |
|  | Zea mays L. | *dhrah*, *dhurah, dhurah shamiya* | Maize fruit | soak in water and drink | Diuretic, urinary retention, urinary tract infections and cystitis, diabetes |
| **Iridaceae** | Iris pallida Lam.  Iris palaestina (Back.) Boiss. | *Sauwsan* | Leaves, rhizome | boil in water and drink | urinary tract infections and retention |
| **Labiatae** | Coridothymus capitatus (L.) Reichb. | *Zahayfy, Za’tar Farsy* | leaves and flowers | boil leaves and flowers in water and drink | urinary tract infections and retention, prostate, testicles pains |
|  | Mentha longifolia L. | *Na’na’ barri, Habaq* | leaves | boil in water and drink | blood in the urine, urinary tract infections and retention, vomiting, nausea |
|  | Micromeria myrtifolia Boiss.et Hohen. | *Zufih* | leaves | boil in water and drink | urinary retention and urinary tract infection. |
|  | Rosmarinus officinalis L. | *Iklil al-Jabal* | leaves | boil the leaves in water and drink | urinary tract infections and retention, vagina infection and sterility |
| **Liliaceae** | Allium ampeloprasum L. | *Thum barri* | fruit, leaves and stalks | mix with olive oil and eat | urinary tract infections and retention, kidney pain |
|  | Asparagus aphyllus L. | *Halyun*, *Hiliun* | leaves | eat as raw salad | diuretic, urinary retention and urinary tract infections |
|  | Asphodelus aestivus Brot. | *Swai, ‘Unsol, 'Onsol* | roots | boil in water and drink | urinary retention and urinary tract infection |
|  | Colchicum ritchii R. Br. | *Wada’, ‘Akna, ‘Ukna, Farj al-Ard* | corms | apply corms in hot aches, peel and eat it | kidney infections, urine pains, and prostate |
|  | Ruscus aculeatus L. | *Ass Barri, Khizana, ‘Urf al-Deik* | roots, leaves | boil in water and drink | urinary tract infections and retention |
| **Linaceae** | Linum pubescens Banks et Sol. | *Kittan* | seeds | boil in water and drink | urinary tract infections, urinary pains, and prostate illnesses |
| **Malvaceae** | Althaea officinalis L. | *khitmiya, khutmiya, Khutmiy* | leaves | eat as raw salad | urinary tract infections and the incontinence |
|  | Lavatera trimestris L. | *Khubbizat al-Far* | leaves, seeds | eat as raw salad, or cook | urinary tract infections, retention and prostate illnesses |
|  | Malva sylvestris L. | *Khubiza, Khubaizih* | leaves | eat as raw salad, or cook, or boil in water and drink | urinary tract infections, retention and prostate illnesses, vaginal infection and skin irritation |
| **Mimosaceae** | Acacia raddiana Savi | *Sayal* | resin | boil in water or milk and drink | urinary retentions and urinary tract infections, and prostate |
|  | Prosopis farcta (Banks et Sol.) | *Yanbut* | leaves, fruit and roots | boil in water and drink | urinary tract infections and retention, diabetes, high blood pressure |
| **Oleaceae** | Olea europaea L. | *Zaytun, Zeit* | oil | drink | urinary retention and urinary tract infection, prostate, kidney pains, skin irritation, constipation |
| **Oxalidaceae** | Oxalis corniculata L. | *Hummaydh* | whole plant | soak in water and drink | urinary retention and urinary tract infection |
| **Fabaceae** | Alhagi maurorum Medik | *'Aqul,‘uqul, Shuk al-Jimal* | rhizome | boil in water and drink | urinary retention and urinary tract infection |
|  | Lupinus albus L. | *Turmus* | seeds, whole herb | soak in water and drink | diuretic, urinary tract infections and retention |
|  | Ononis antiquorum L. | *Shibriq Shaik* | leaves, flowers and dried roots | boil in water and drink | diuretic, urinary tract infections and retention |
|  | Scorpiurus muricatus L. | *lisan al-Kalb* | leaves, stems and stalks | eat as raw salad | urinary tract infections |
|  | Trigonella foenum-graecum L. | *Hilbih* | seeds | boil seeds in water and drink | urinary tract infections and retention, diabetes, and high blood pressure |
| **Pinaceae** | Pinus halepensis Mill. | *Snubar, Qriesh* | leaves | boil in water and drink | urinary retentions, cystitis and prostate illnesses |
| **Plantaginaceae** | Plantago lagopus L. | *Yanama, ‘Ishbit al-Baraghith* | leaves | boil in water and drink | urinary tract infections |
| **Polygonaceae** | Polygonum equisetiforme Sm. | *Qudhdhab* | leaves | boil in water and drink | urinary retention and urinary tract infections, blood in the urine. |
|  | Rheum palaestinum Feinbr. | ‘*Uttrafan* | leaves and roots | boil in water and drink | urinary retention and urinary tract infections. |
|  | Rumex cyprius Murb. | *Hummidhah, Hummidh* | leaves | eat as raw salad | urinary tract infections and retentions, blood in the urine |
| **Portulacaceae** | Portulaca oleracea L. | *Farfahina, Rijlih* | leaves and stalks | crush, soak in water and drink, or eat as raw salad | diuretic, urinary tract infections and retention, cystitis |
| **Ranunculaceae** | Adonis aestivalis L. | *‘Ain al-Deik* | leaves | soak in water and drink | urinary tract infections, prostate, impotency |
|  | Nigella sativa L. | *Habbit al-Barakah, Habbih Suda, Qazhih* | seeds, or extracted oil | boil the seeds in water and drink, or drink the oil | urinary tract infections and retention, prostate illnesses, blood in urine, diabetes, impotency, sterility |
| **Rhamnaceae** | Zizyphus spina-christi (L.) Desf. | *Sidr, Dowm* | fruit, or leaves | eat the fruit, soak leaves in water and drink | urinary tract infections and prostate |
| **Rosaceae** | Rubus sanctus Schreb. | *‘Ullayq* | berries, or leaves | eat the berries, soak leaves in water and drink | urinary retention and prostate illnesses |
| **Rubiaceae** | Rubia tenuifolia D’Urv. | *Fuwwa* | roots | boil in water and drink | diuretic, urinary retentions, skin irritation |
| **Rutaceae** | Citrus limon L. | *lamun* | fruit | drink with water or tea | urinary tract infections and retention, stomach ach, diarrhea, upset, nausea |
| **Solanaceae** | Mandragora autumnalis Bertol. | *Mjininih, Yabruh, Tuffah al-Majanin* | ripe fruit | eat | urinary tract infections, sterility, vaginal infections, increasing breast milk, evil eye |
| **Scrophulariaceae** | Linaria cymbalaria Mill. | *Hashishat al-Shuquq* | leaves | boil in water and drink | diuretic, urinary tract infections and retention, breast infection |
| **Umbelliferae** | Anisum vulgare L. | *Yansun* | seeds | soak or boiled in water and drink | diuretic, urinary tract infections and retention |
|  | Apium graveolens L. | *Karafs* | leaves, roots | boil in water and drink | urinary tract infections and pains, incontinence and retention |
| **Umbelliferae** | Conium maculatum L. | *Shiqran, Sikran, shoukaran* | dried leaves | soak in water and drink | urinary tract infections and prostate illnesses |
|  | Petroselinum crispum/  Petroselinum sativum | *Baqdunis* | leaves | soak in water and drink | urinary tract infections and retention, and prostate illnesses |
|  | Pituranthos tortuosus (Desf) Benth.ex Aschers.et Schweinf. | *Zaqquh, Zagguh* | stems and flowers | boil in water and drink | blood in the urine, urinary retention |
| **Urticaceae** | Parietaria judaica L. | *Kuzziza* | leaves | Boil in water and drink | urinary tract infections, urinary retention, vaginal infection |
|  | Urtica pilulifera/ Urtica urens | *Qurris*, *Hurriq* | leaves | Boil in water and drink | urinary tract infection and retention |
| **Zygophyllaceae** | Tribulus terrestris L. | *Draiseh, Qatab* | dried leaves | Boil in water and drink | urinary tract infections and retention |
